# Molecular Pathogenesis and Targeted Therapies in Eosinophilic Granulomatosis with Polyangiitis: An Updated Review

**DOI:** 10.3390/ijms262211141

**Published:** 2025-11-18

**Authors:** María López Paraja, Grisell Starita Fajardo, Ignacio Donate Velasco, David Lucena López, María Pilar Iranzo Alcolea, Francisco José Lirola Sánchez, Mercedes Peña Rodriguez, Andrés González García, Luis Manzano Espinosa

**Affiliations:** 1 Systemic Autoinmmune Diseases Unit, Department of Internal Medicine, Hospital Universitario Ramón y Cajal, Instituto Ramón y Cajal de Investigación Sanitaria (IRYCIS), 28034 Madrid, Spain; mlparaja@salud.madrid.org (M.L.P.); grisell.starita@salud.madrid.org (G.S.F.); ignacio.donate@salud.madrid.org (I.D.V.); davidlulo95@gmail.com (D.L.L.); mp.iranzoalcolea@gmail.com (M.P.I.A.); franlirolas@gmail.com (F.J.L.S.); merprazpi@gmail.com (M.P.R.); luis.manzano@uah.es (L.M.E.); 2 Faculty of Medicina and Health Sciences, Universidad de Alcalá (UAH), 28801 Alcalá de Henares, Spain

**Keywords:** eosinophilic granulomatosis with polyangiitis, ANCA-associated vasculitis type 2 inflammation, eosinophils, IL-5, biomarkers, targeted therapy

## Abstract

Eosinophilic granulomatosis with polyangiitis (EGPA) is a rare systemic vasculitis characterized by asthma, eosinophilia, and necrotizing inflammation of small- to medium-sized vessels. Accumulating evidence indicates that EGPA is a polygenic and heterogeneous disorder comprising distinct antineutrophil cytoplasmic antibody (ANCA)–defined endotypes with divergent genetic backgrounds, immune pathways, and clinical phenotypes. Its pathogenesis reflects the convergence of epithelial–alarmin signaling, type 2 inflammation, eosinophil effector mechanisms, and B-cell/autoantibody responses, with myeloperoxidase (MPO)-ANCA serving as a hallmark of the vasculitic subset. Recent advances in genomics, immunology, and multi-omics profiling have uncovered biomarkers and molecular circuits sustaining disease activity and guiding therapeutic stratification. The identification of the interleukin (IL)-5–eosinophil axis, epithelial-derived alarmins, and B-cell/IgG4 networks as central pathogenic nodes has enabled the development of targeted biologic therapies that are redefining treatment paradigms. Benralizumab (anti-IL-5Rα) has recently been approved for EGPA following the phase 3 head-to-head MANDARA trial, which demonstrated non-inferiority to mepolizumab in achieving remission (BVAS = 0 with ≤4 mg/day prednisone equivalent) at weeks 36 and 48. These results, together with the established efficacy of mepolizumab, inform practical selection between IL-5 and IL-5Rα blockade and support glucocorticoid-sparing approaches. A structured literature search (2015–2025) was conducted in PubMed, Scopus, and Web of Science to identify recent advances in epidemiology, genetics, biomarkers, and targeted therapies for EGPA. This updated review integrates molecular insights, clinical endotypes, and therapeutic innovations to outline current evidence and future precision-medicine strategies aimed at improving long-term patient outcomes.

## 1. Introduction

EGPA is a rare systemic vasculitis characterized by eosinophil-rich and necrotizing inflammation of small- to medium-sized vessels, most commonly associated with asthma and peripheral eosinophilia [1,2,3]. It belongs to the spectrum of ANCA-associated vasculitides (AAV), although ANCA positivity, predominantly directed against MPO, is detected in only 30–40% of patients [4,5,6].

Recent population-based studies have refined epidemiologic estimates, reporting an incidence of approximately 1–4 cases per million person-years and a prevalence ranging from 10 to 46 per million, with notable geographic variability in clinical presentation and ascertainment [7,8,9]. The introduction of the 2022 ACR/EULAR classification criteria has enabled more standardized case identification and improved comparability across studies [10].

Despite its rarity, EGPA poses significant diagnostic and therapeutic challenges owing to its dual pathobiology, eosinophil-driven inflammation and ANCA-mediated vasculitis, which contribute to its diverse organ involvement and variable disease course [6,11,12,13]. Advances in immunopathogenesis have elucidated key molecular pathways involving IL-5, the IL-5 receptor-α (IL-5Rα), epithelial alarmins, and B-cell-mediated immune responses, paving the way for the development of targeted biologic therapies [14,15,16,17].

This review summarizes recent advances in the molecular mechanisms, clinical phenotypes, biomarkers, and emerging targeted therapies of EGPA, integrating data from 2015 to 2025 and highlighting precision-medicine perspectives aimed at improving long-term outcomes.

## 2. Methods

This review was conducted as a structured narrative synthesis in accordance with current recommendations for evidence-based clinical reviews. A comprehensive literature search was performed in PubMed, Scopus, and Web of Science covering the period from January 2015 to April 2025, using combinations of the following keywords: “eosinophilic granulomatosis with polyangiitis,” “Churg–Strauss syndrome,” “ANCA-associated vasculitis,” “biomarkers,” “genetics,” “treatment,” and “clinical phenotypes.” Additional references were identified through manual screening of bibliographies and recent consensus statements.

Inclusion criteria comprised peer-reviewed human studies, meta-analyses, clinical trials, cohort studies, and major reviews addressing the pathogenesis, clinical features, biomarkers, or management of EGPA. Exclusion criteria included non-English publications, isolated case reports, and animal or in vitro studies not directly related to disease mechanisms.

The available evidence was qualitatively appraised according to study design and level of evidence, prioritizing randomized controlled trials, population-based cohorts, and expert consensus. Where applicable, international guidelines (EULAR, ACR, and EUVAS) were referenced to contextualize therapeutic recommendations.

This review integrates genetic, immunopathogenic, and clinical perspectives to provide an updated synthesis of the current understanding and emerging therapeutic approaches in EGPA.

## 3. Epidemiology

EGPA is a rare disease. Contemporary population-based cohorts and systematic reviews report an incidence of approximately 1–4 cases per million person-years and a prevalence of 10–46 per million in European populations, with an apparent rise in recorded prevalence over time that likely reflects improved recognition, broader diagnostic criteria, and enhanced survival [7,8,9].

The age at diagnosis typically clusters in middle adulthood (40–60 years), and most large cohorts show no consistent sex predominance [7,8,9]. Geographic variation exists: incidence and prevalence rates appear higher in European and North American populations compared with earlier Asian series, although recent nationwide data from Korea and Japan indicate that the disease occurs worldwide with a comparable age distribution [7,8]. Given its rarity, precise racial or ethnic comparisons remain limited and are influenced by case ascertainment methods and healthcare access [7].

There is a strong association with asthma and related medications. Asthma occurs in more than 90% of patients and usually precedes systemic vasculitis by several years; chronic rhinosinusitis with nasal polyps (CRSwNP) is also common [4,5,6]. The potential relationship between leukotriene receptor antagonists (LTRAs), particularly montelukast, and EGPA remains debated. Current evidence suggests that these agents may unmask latent eosinophilic vasculitis during corticosteroid tapering rather than directly causing the disease [18,19]. This association should therefore be regarded as observational and hypothesis-generating, emphasizing the need for careful clinical interpretation.

## 4. Etiopathogenesis

EGPA is a polygenic disorder comprising two principal ANCA-defined endotypes with distinct immunogenetic signatures. The landmark genome-wide association study (GWAS) by the European Vasculitis Genetics Consortium provided the first molecular evidence that the disease represents two biologically distinct entities, separating MPO-ANCA–positive from ANCA-negative forms [11,12,13].

In MPO-ANCA–positive EGPA, susceptibility is primarily driven by HLA-DQ loci, confirming its overlap with microscopic polyangiitis and supporting a classical autoimmune vasculitic background.

In contrast, the ANCA-negative subset is associated with non-HLA variants involved in mucosal biology and eosinophil regulation, notably TSLP, BCL2L11, CDK6, BACH2, LPP, GPA33, and IRF1/IL5. These loci converge on pathways regulating epithelial–immune crosstalk, type 2 inflammation, and eosinophil survival.

Several of these variants (TSLP, BCL2L11, IL5, IRF1) are shared with severe asthma and peripheral eosinophil count traits, reinforcing the concept that the eosinophilic form of EGPA lies within a broader allergic/type 2 inflammatory spectrum. Among these, TSLP underscores the role of the airway epithelium as an initiator of type 2 immunity via alarmin-mediated activation of Th2 and group 2 innate lymphoid cells (ILC2), whereas BCL2L11 and BACH2 implicate defective lymphoid apoptosis and dysregulated Th2/Th17 balance, respectively.

Exploratory signals at CDK6 and LPP may contribute to aberrant cell-cycle and adhesion dynamics, facilitating leukocyte migration into affected tissues. Although these findings require replication, they collectively support a unifying model in which epithelial barrier dysfunction, eosinophil persistence, and lymphoid dysregulation converge beyond the classical IL-5–eosinophil axis.

**Figure 1:**
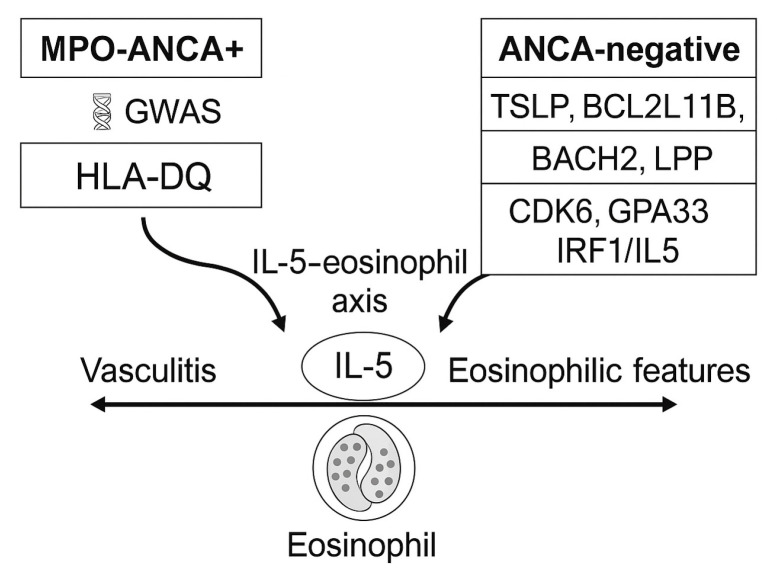
Genetic endotypes in eosinophilic granulomatosis with polyangiitis (EGPA). The disease segregates into two principal antineutrophil cytoplasmic antibody (ANCA)–defined endotypes: myeloperoxidase (MPO)-ANCA–positive EGPA, associated with HLA-DQ–restricted autoimmunity and a vasculitic phenotype, and ANCA-negative EGPA, linked to non-HLA variants (TSLP, BCL2L11, BACH2, LPP, CDK6, GPA33, IRF1/IL5) driving eosinophilic inflammation and mucosal immune activation. Both converge on the IL-5–eosinophil axis as a shared downstream pathway. Abbreviations: ANCA, antineutrophil cytoplasmic antibody; MPO, myeloperoxidase; IL, interleukin; HLA, human leukocyte antigen; EGPA, eosinophilic granulomatosis with polyangiitis.

**Figure 2:**
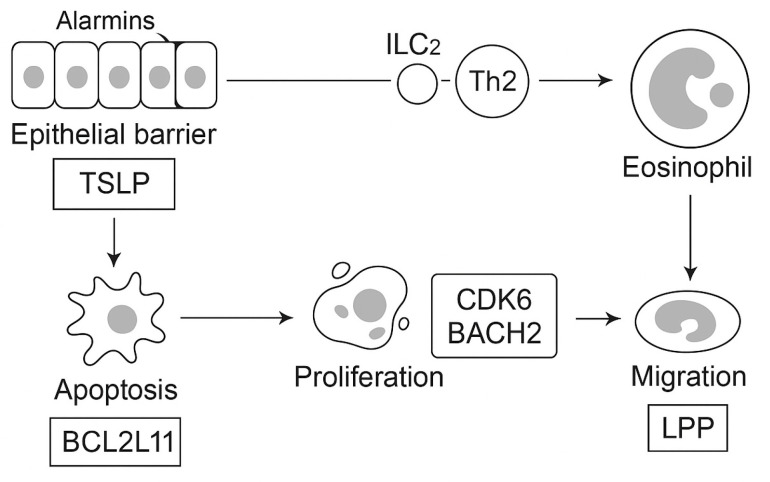
Epithelial barrier cells release alarmins such as thymic stromal lymphopoietin (TSLP), which activate group 2 innate lymphoid cells (ILC2) and type 2 helper T cells (Th2), driving eosinophil proliferation and activation. Genetic variants in BCL2L11 regulate apoptosis resistance, CDK6 and BACH2 influence lymphocyte proliferation and differentiation, and LPP facilitates eosinophil migration. Additional loci, GPA33 and IRF1/IL5, identified in genome-wide association studies (GWAS), are shared with severe asthma and eosinophil count traits. Together, these networks converge on the IL-5–eosinophil axis, linking epithelial dysfunction, lymphoid dysregulation, and tissue infiltration as key molecular pathways in EGPA. Abbreviations: EGPA, eosinophilic granulomatosis with polyangiitis; ILC2, group 2 innate lymphoid cell; Th2, type 2 helper T cell; IL, interleukin; GWAS, genome-wide association study; TSLP, thymic stromal lymphopoietin.

The genetic and endotype architecture of EGPA, including key HLA-DQ and non-HLA susceptibility loci identified in GWAS, is summarized in Figure 1. Key elements of immune dysregulation and effector interplay are depicted in Figure 2.

**Immune dysregulation and effector pathways**

Building on the genetic framework, the immunopathogenesis of EGPA unfolds within a type 2–dominated inflammatory milieu. Epithelial alarmins such as thymic stromal lymphopoietin (TSLP), interleukin (IL)-33, and IL-25 amplify Th2 and ILC2 responses, promoting the production of IL-5 and IL-13, systemic eosinophilia, and eotaxin-mediated tissue recruitment (CCL11, CCL24, CCL26) [14,15,20]. These cascades explain the strong respiratory tropism of the disease as well as the eosinophilic cardiac and gastrointestinal involvement characteristic of ANCA-negative EGPA [9,14].

In parallel, the humoral compartment is characterized by the coexistence of anti-MPO ANCA, particularly in the vasculitic endotype, and increased IgG4 production, reflecting chronic Th2 polarization [7,11,17]. Together, these features underscore the dual pathogenic architecture of EGPA, where eosinophil-driven inflammation and B-cell/autoantibody-mediated autoimmunity variably coexist across patients.

**Figure 3:**
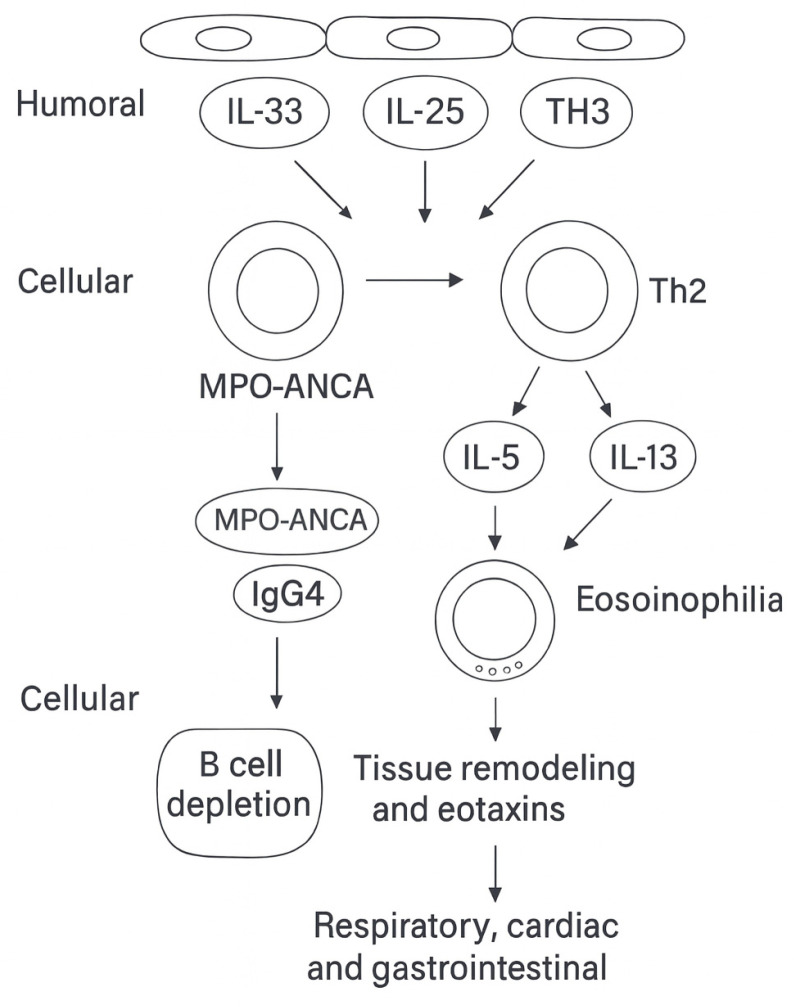
Immune dysregulation and effector pathways in eosinophilic granulomatosis with polyangiitis (EGPA). Epithelial alarmins—thymic stromal lymphopoietin (TSLP), interleukin (IL)-33, and IL-25—activate group 2 innate lymphoid cells (ILC2) and type 2 helper T cells (Th2), amplifying type 2 immunity through the production of IL-5 and IL-13, systemic eosinophilia, tissue remodeling, and eotaxin-mediated chemotaxis (CCL11, CCL24, CCL26). These cascades are thought to underlie the respiratory tropism and the eosinophilic cardiac and gastrointestinal involvement typical of antineutrophil cytoplasmic antibody (ANCA)–negative EGPA. In parallel, the humoral compartment is characterized by myeloperoxidase (MPO)-ANCA, particularly in the vasculitic endotype, and increased immunoglobulin G4 (IgG4)production, reflecting Th2 polarization and linking to B-cell dysregulation [7,9]. Abbreviations: EGPA, eosinophilic granulomatosis with polyangiitis; ANCA, antineutrophil cytoplasmic antibody; MPO, myeloperoxidase; IL, interleukin; ILC2, group 2 innate lymphoid cell; Th2, type 2 helper T cell; IgG4, immunoglobulin G4.

A schematic representation of immune dysregulation and effector interactions is provided in Figure 3.

**Critical appraisal of current biology**

The pathogenic contribution of anti-MPO ANCA remains debated and likely context-dependent. Rather than acting as a universal trigger, ANCA may function as a disease amplifier in subsets with overt small-vessel vasculitis [16,18]. Likewise, the evidence supporting eosinophil extracellular trap formation (EETosis) in EGPA, while compelling, derives from small, histology-centered cohorts. In vivo kinetics, reproducibility, and standardized assays for EET-derived markers are not yet established, precluding their use for clinical monitoring [19,20]. Similarly, candidate serum biomarkers such as eosinophil granule proteins (ECP, EDN), TARC/CCL17, and alarmins correlate with disease activity in observational studies but lack external validation, analytical harmonization, and threshold standardization [21,22,23]. They should therefore complement, not replace, BVAS-based assessment and organ-specific evaluation.

**Eosinophil effector mechanisms**

Eosinophils are the principal effector cells in EGPA and mediate tissue damage through multiple mechanisms. Classical degranulation releases cytotoxic proteins—ECP, EDN, eosinophil peroxidase (EPO), and major basic protein (MBP)—which contribute to endothelial injury, fibrosis, and microvascular damage [14,15].

EETosis, an alternative form of eosinophil cell death, has been demonstrated in skin and vascular lesions from limited patient cohorts, correlating with tissue eosinophilia and clinical activity [14,15,16]. Both “suicidal” and “vital” EETosis variants have been described, potentially sustaining chronic inflammation and fibrosis. However, evidence remains largely observational, and assay standardization is lacking, restricting its translation into biomarkers or therapeutic targets.

Experimental data also implicate eosinophils in thromboinflammatory processes, interacting with endothelial and platelet compartments to promote vascular injury and cardiac complications [16]. Collectively, these pathways highlight eosinophils as central orchestrators of vascular inflammation and tissue remodeling, beyond their traditional cytotoxic role, and provide the biological rationale for searching for circulating eosinophil-derived biomarkers reflecting disease activity and prognosis.

**B-cell/IgG4 networks and ANCA**

Beyond type 2 inflammation, B-cell dysregulation represents a central pathogenic axis in EGPA. The presence of anti-MPO ANCA, confined mainly to the vasculitic endotype, supports an autoantibody-mediated component responsible for glomerulonephritis, neuropathy, and purpura [6,7].

Concomitant IgG4 elevation, a marker of Th2-skewed immune activation, has been consistently reported in EGPA but does not imply IgG4-related disease, underscoring the need to avoid diagnostic conflation [14]. The pathogenic relevance of B cells is further supported by the clinical efficacy of rituximab, particularly in ANCA-positive patients, where disease manifestations parallel those of microscopic polyangiitis [17]. Conversely, ANCA-negative EGPA relies predominantly on eosinophil-mediated and barrier-driven mechanisms, aligning with its cardiac and gastrointestinal tropism [7,9].

Altogether, these findings emphasize the heterogeneous contribution of B-cell immunity across EGPA endotypes and its therapeutic implications.

**Barrier biology and exposome**

The airway epithelium serves as both a physical and immunologic interface, releasing alarmins (TSLP, IL-33, IL-25) that activate Th2 and ILC2 cells and promote eosinophil recruitment via eotaxins [14,15,20]. These epithelial signals bridge genetic susceptibility with environmental exposures, explaining the predominance of asthma and chronic rhinosinusitis as sentinel features.

Epidemiologic studies have linked silica exposure, organic solvents, and farming activities with an increased risk of EGPA, whereas cigarette smoking has paradoxically been associated with a reduced risk [18,19]. These associations remain hypothesis-generating, with mechanisms yet to be elucidated. It is plausible that such exposures converge on epithelial stress responses and type 2 cytokine release, amplifying eosinophilic inflammation [9].

Thus, the epithelium emerges not only as a barrier but as an active immunologic hub integrating genetic, microbial, and environmental cues. This concept underscores the significance of gene–environment interactions and highlights epithelial–alarmin pathways as potential upstream therapeutic targets in EGPA [14,15,20].

## 5. Clinical Phenotypes, Biomarkers, and Conventional Therapy

### 5.1. Endotype-Guided Clinical Features and Organ Risk

EGPA encompasses two major ANCA-defined endotypes with distinct biological and clinical trajectories. ANCA-positive EGPA (usually MPO-ANCA) follows a vasculitic course characterized by small-vessel inflammation leading to glomerulonephritis, mononeuritis multiplex, alveolar hemorrhage, and purpura. These patients display a higher relapse propensity and require close, long-term follow-up [6,8,10].

In contrast, ANCA-negative EGPA is dominated by eosinophil-mediated pathology, including eosinophilic myocarditis or cardiomyopathy, pulmonary infiltrates, and gastrointestinal involvement. Although relapses tend to be less frequent, cumulative cardiac damage remains a major determinant of prognosis [6,7]. Asthma (present in more than 90% of patients) and chronic rhinosinusitis with nasal polyps occur across both subsets [9]. A comparative overview of the two principal endotypes is shown in Table 1.

Overlap between endotypes is common (e.g., MPO-ANCA positivity with eosinophilic myocarditis, or ANCA-negative disease with neuropathy). Such mixed patterns reflect converging biology—type 2 inflammation plus humoral autoimmunity—and often require stepwise or combined therapy: treat vasculitis first (rituximab ± cyclophosphamide) when organ-threatening, and add IL-5/IL-5Rα blockade for eosinophil-driven control and glucocorticoid sparing [16,23].

Cardiac involvement is the leading predictor of morbidity and mortality, particularly in ANCA-negative EGPA. Baseline and follow-up evaluation should include ECG and transthoracic echocardiography in all patients. Cardiac MRI is recommended in the presence of symptoms, biomarker elevation, or unexplained ECG changes, and should be periodically repeated even in clinically stable cases [7,23,24].

### 5.2. Biomarker Signatures by Endotype

In ANCA-positive EGPA, MPO-ANCA serves as a diagnostic and prognostic marker of vasculitic involvement, although titers may not consistently reflect disease activity. These patients typically exhibit lower peripheral eosinophil counts than ANCA-negative cases [25,26].

ANCA-negative EGPA displays a stronger type 2 inflammatory profile, with elevated IL-5 levels and prominent tissue eosinophilia. Candidate biomarkers, including TARC/CCL17, ECP, EDN, and EET-derived cell-free DNA, correlate with inflammatory activity in small studies but remain exploratory and unvalidated [11,13,21,22]. IgG4 elevation is frequently observed across both subsets and reflects chronic Th2 polarization rather than IgG4-related disease. Proteomic and multi-omic signatures involving serum amyloid A 1 (SAA1), fibrinogen alpha chain (FGA), serum amyloid P component (SAP), and cholesteryl ester transfer protein (CETP) are under evaluation for distinguishing active disease from remission but require external validation before routine use [22,23].

**Figure 4:**
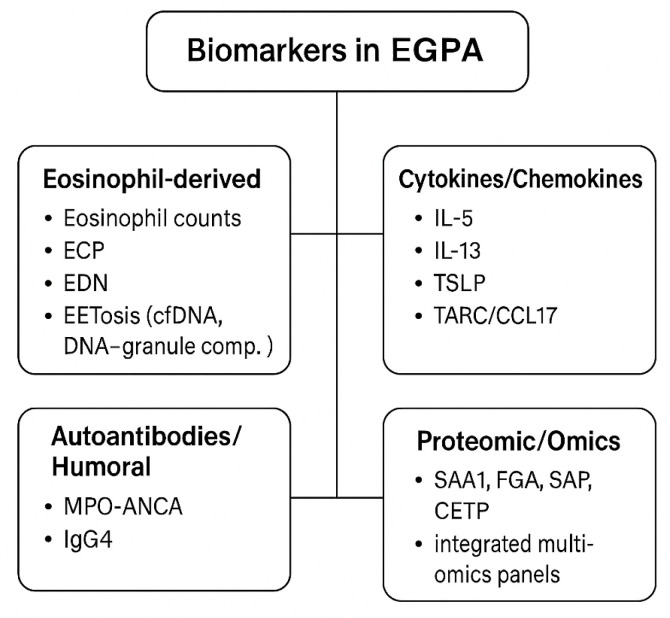
Biomarkers in EGPA. Candidate biomarkers in EGPA can be stratified into four major categories: eosinophil-derived markers, including peripheral eosinophil counts, eosinophil cationic protein (ECP), eosinophil-derived neurotoxin (EDN), and markers of eosinophil extracellular trap cell death (EETosis), such as cell-free DNA and DNA–granule complex; cytokines and chemokines, with type 2 cytokines (IL-5, IL-13), epithelial alarmins (TSLP, IL-33), and TARC/CCL17 as potential activity markers; autoantibodies and humoral markers, mainly MPO-ANCA, which is associated with the vasculitic endotype, and increased IgG4 levels reflecting Th2 skewing; and proteomic and multi-omic candidates, such as serum amyloid A1 (SAA1), fibrinogen alpha chain (FGA), serum amyloid P (SAP), cholesteryl ester transfer protein (CETP), and integrated multi-omics panels under evaluation [6,14,21,22] All remain investigational, and none currently replace BVAS-based activity scoring or organ-specific imaging in clinical practice.

At present, clinical endotyping relies primarily on ANCA status, eosinophil count, and phenotype [13,16]. TARC/CCL17 and EET-related assays show promise but require assay standardization and defined thresholds [19,21]. Proteomic and multi-omic profiles can distinguish activity from remission in research settings but are not yet suitable for clinical triage [22]. Priorities for translation include harmonized assay platforms, validated cut-offs, and prospective correlation with BVAS-based outcomes. Candidate biomarker domains are summarized in Figure 4.

### 5.3. Conventional Therapy, Sequencing, and Maintenance

**Evidence base and trial design**

Compared with other AAVs, the evidence base for EGPA remains limited [3,23]. The MIRRA trial (mepolizumab versus placebo) is the only pivotal randomized controlled trial to date [27], while MANDARA (benralizumab versus mepolizumab) provided the first head-to-head biologic comparison [28]. Most remaining data derive from observational cohorts, single-arm studies, or extrapolation from GPA and MPA [16,22,26]. This heterogeneity mandates individualized, endotype-aware management and cautious generalization beyond studied populations, particularly in organ- or life-threatening vasculitic presentations.

**Induction therapy**

Glucocorticoids remain the mainstay of induction therapy (0.5–1 mg/kg/day prednisone equivalent), tapered over 6–12 months according to disease severity and organ involvement. Relapse prevention and mitigation of steroid toxicity—through bone protection, infection prophylaxis, and cardiovascular risk control—are essential [3,23].

In organ- or life-threatening vasculitic disease, particularly ANCA-positive EGPA, cyclophosphamide or rituximab is recommended for induction [23,29]. Cyclophosphamide is preferred when rapid cytoreduction is required (e.g., glomerulonephritis, alveolar hemorrhage), whereas rituximab is favored in relapsing disease or when alkylating agents are contraindicated. Maintenance is usually achieved with rituximab, azathioprine, methotrexate, or mycophenolate mofetil, depending on tolerance, comorbidities, and reproductive considerations [29].

**Biologic therapy**

In eosinophil-dominant disease (typically ANCA-negative), targeting the IL-5 axis has transformed management. Mepolizumab (300 mg subcutaneous every 4 weeks) achieved significant glucocorticoid-sparing effects in MIRRA [27], while benralizumab (30 mg subcutaneous every 4 weeks) was non-inferior to mepolizumab in MANDARA, with comparable remission and steroid-sparing outcomes [28]. Reslizumab remains an alternative for selected patients [30]. Both MIRRA and MANDARA defined remission as BVAS = 0 with prednisone ≤4 mg/day, establishing standardized criteria for relapse and glucocorticoid-sparing evaluation [27,28].

In airway-predominant disease (asthma ± CRSwNP), IL-4Rα blockade (dupilumab) may improve airway control, though caution is warranted in ANCA-positive phenotypes due to reported vasculitic flares. Upstream alarmin blockade (tezepelumab) is currently investigational [23,31,32].

Real-world practice increasingly integrates biologic switching (IL-5 → IL-5Rα → B-cell-directed) or combination strategies to achieve sustained remission [16,23,28]. Biologics should complement, not replace, comprehensive monitoring and organ-specific management.

**Maintenance and sequencing**

Maintenance therapy aims for durable remission with the lowest effective corticosteroid dose [3,23].

Vasculitic/ANCA-positive: prioritize B-cell-directed therapy (rituximab ± cyclophosphamide), followed by maintenance immunosuppression [3,23,29,33]

Eosinophilic/ANCA-negative: favor IL-5 or IL-5Rα blockade for remission induction and tapering [27,29,30,34]

Airway-predominant: combine IL-5 or IL-4Rα biologic with conventional vasculitis therapy as indicated [23,27,28,31]

Sequential or switch strategies (e.g., IL-5 → IL-5Rα or biologic → rituximab) may be considered when control is suboptimal. Conventional immunosuppressants remain valuable maintenance options in resource-limited settings or when biologics are contraindicated [15,23,26,28]

An overview of conventional and biologic agents, their mechanisms, and clinical indications in EGPA is summarized in Table 2.

**Glucocorticoid tapering and toxicity mitigation**

Glucocorticoids remain indispensable but contribute substantially to morbidity. After disease control, taper gradually over 3–6 months, aiming for ≤5 mg/day by 6–12 months when feasible [3,23]. Faster tapering is appropriate when effective biologic therapy is established, while slower reduction is warranted in cardiac disease or delayed remission.

Toxicity prevention measures include bone protection, Pneumocystis prophylaxis, metabolic and cardiovascular monitoring, and vaccination (influenza, pneumococcus, herpes zoster) according to CDC and EULAR recommendations [3,23]. A structured tapering plan with early introduction of steroid-sparing agents is central to long-term management.

**Safety considerations**

Biologic therapies are generally well tolerated, though vigilance is required for herpes zoster reactivation, helminth infection, and hypersensitivity reactions [3,23]. Vaccination status should be optimized before immunosuppression, and live vaccines avoided during therapy [3,23]. Perioperative planning should coordinate biologic dosing intervals to minimize infection risk and ensure safe surgical timing.

Despite substantial therapeutic progress, sustained steroid-free remission beyond 12–18 months is achieved in fewer than half of patients, highlighting the unmet need for biomarker-guided precision strategies [23,28]

**Precision-therapy perspective**

Aligning treatment with the dominant immunopathogenic signature represents the next step toward precision medicine in EGPA.

Vasculitic/ANCA-positive: prioritize B-cell-directed therapy (rituximab ± cyclophosphamide); IL-5 or IL-5Rα blockade may be added for eosinophilia and asthma control.

Eosinophilic/ANCA-negative: favor IL-5 or IL-5Rα inhibition, with escalation to IL-5Rα agents (e.g., benralizumab) in patients with persistent eosinophilia, relapse after IL-5 therapy, or preference for single-dose administration every 4 weeks.

Refractory or mixed phenotypes: consider enrollment in clinical trials evaluating novel approaches such as JAK inhibitors, anti-Siglec-8 antibodies, or plasma-cell-targeting agents (e.g., daratumumab). Ongoing research integrating molecular, immunologic, and multi-omic profiling aims to refine patient stratification, guide therapeutic sequencing, and ultimately enable adaptive, biomarker-driven treatment algorithms [23,28,32,36,37].

## 6. Molecular Therapeutic Targets and Emerging Treatments in EGPA

**IL-5 axis**

The IL-5 pathway represents the best-validated molecular target in EGPA, as demonstrated by the success of anti-IL-5 and anti-IL-5Rα therapies now integrated into routine care [23,27,28]. Mepolizumab (anti-IL-5) was the first biologic approved for EGPA following the pivotal MIRRA trial, which showed a significantly longer duration of remission and reduced glucocorticoid dependence compared with placebo [23,27].

Subsequently, benralizumab (anti-IL-5Rα) received regulatory approval for adult EGPA after the phase 3 MANDARA trial MANDARA was a 52-week, double-blind, active-controlled study comparing benralizumab 30 mg subcutaneously every 4 weeks with mepolizumab 300 mg subcutaneously every 4 weeks. Benralizumab achieved non-inferior remission rates at weeks 36 and 48 (59% vs. 56%; difference 3%, 95% CI −13 to 18), with comparable safety and steroid-sparing effects. Remission was defined as BVAS = 0 and prednisone ≤ 4 mg/day [28].

Mechanistically, benralizumab binds IL-5Rα, inducing antibody-dependent eosinophil depletion and providing faster and deeper eosinophil clearance in patients with high baseline counts or refractory disease. Mepolizumab remains the benchmark agent with the most extensive real-world evidence base, while reslizumab is an alternative in selected cases [23,27,30].

**Figure 5:**
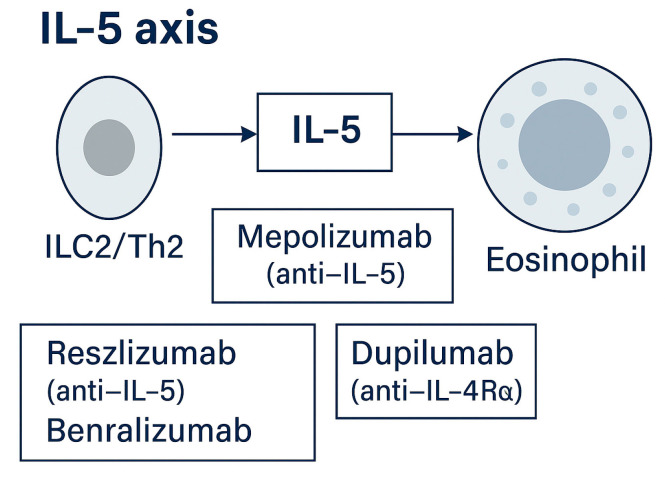
IL-5 axis. ILC2/Th2 cells produce IL-5, promoting eosinophil maturation and survival. Targeted therapies include mepolizumab and reslizumab (anti-IL-5) and benralizumab (anti-IL-5Rα) [17,23,27,28,30]. Approved agents include mepolizumab (anti-IL-5) and benralizumab (anti-IL-5Rα: approved for EGPA based on MANDARA): reslizumab is used in selected off-label scenarios [30,36].

These results firmly establish the IL-5–eosinophil axis as a cornerstone of EGPA pathogenesis and a paradigm for biologic precision therapy. The IL-5 pathway and associated targets are summarized in Figure 5 [23,27,30].

**IL-4/IL-13 axis and epithelial–alarmin targeting**

Type 2 inflammation in EGPA is further driven by IL-4 and IL-13, which signal through the shared IL-4Rα receptor. Dupilumab, a monoclonal antibody blocking IL-4Rα, inhibits both cytokines and has proven efficacy in severe asthma and chronic rhinosinusitis with nasal polyps (CRSwNP) [31]. Small case series and observational studies suggest benefit in EGPA, particularly for refractory airway disease, although available data remain limited and heterogeneous [15,23].

Importantly, several reports have described EGPA onset or vasculitic flare shortly after dupilumab initiation in patients with severe asthma. Accordingly, dupilumab should be regarded as an adjunct for airway control rather than a primary vasculitis-directed therapy, and its use warrants multidisciplinary monitoring, especially in ANCA-positive subsets [15,23,31].

Upstream of IL-4/IL-13 signaling, epithelial-derived alarmins such as TSLP, IL-33, and IL-25 initiate eosinophilic inflammation. Tezepelumab (anti-TSLP) has demonstrated broad efficacy in severe asthma irrespective of eosinophil counts, but its role in EGPA remains investigational [32]. Early-phase studies targeting IL-33 or IL-25 may offer future strategies to modulate barrier-driven inflammation, particularly in ANCA-negative, eosinophil-dominant subsets [17].

**Figure 6:**
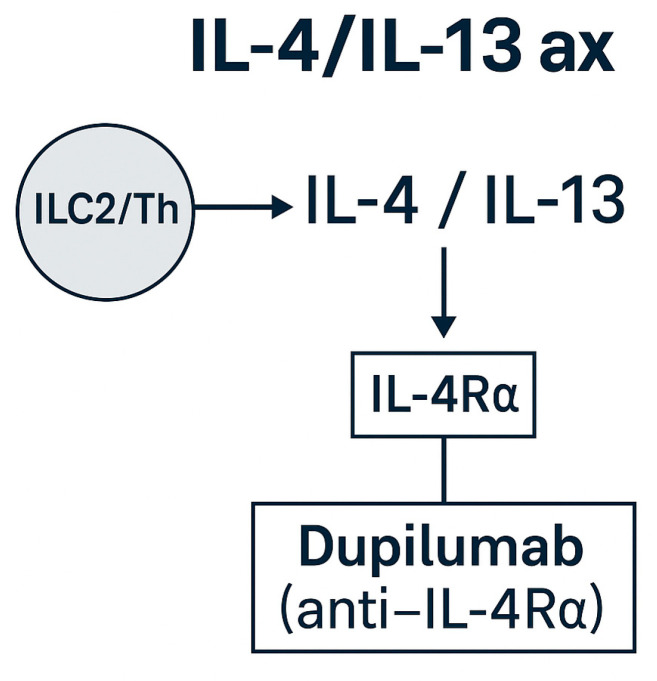
IL-4/IL-13 and epithelial–alarmin axes in EGPA. Schematic representation of IL-4/IL-13 signaling through IL-4Rα and the upstream epithelial alarmins (TSLP, IL-33, IL-25) that drive type 2 inflammation. Dupilumab (anti-IL-4Rα) is approved for asthma/CRSwNP and may benefit airway-dominant EGPA [15,17,23,31]: tezepelumab (anti-TSLP) and anti-IL-33/IL-25 agents remain investigational [17,31].

**Figure 7:**
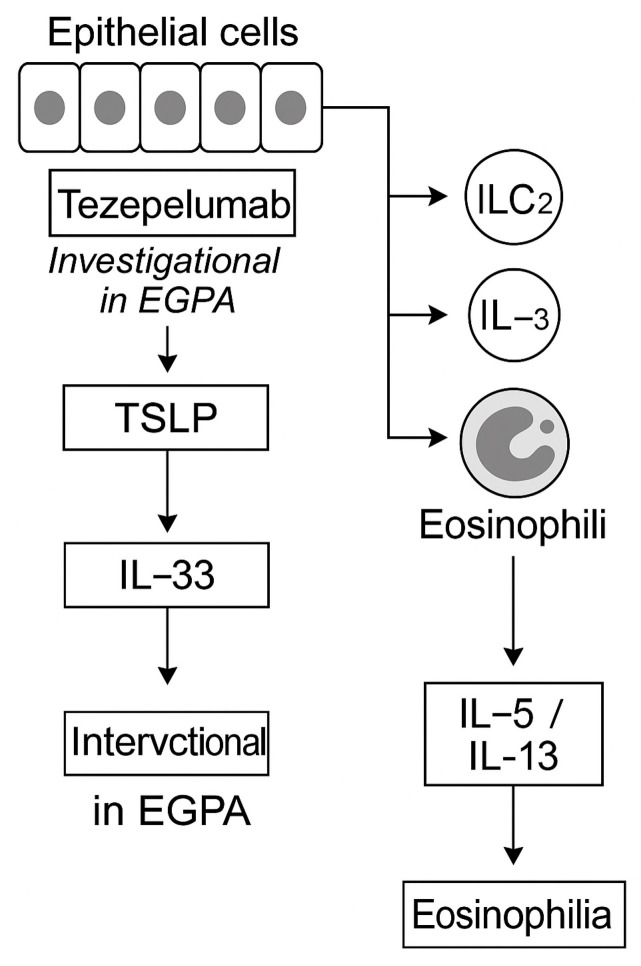
Epithelial alarmins and upstream blockade in EGPA. Epithelial-derived alarmins, thymic stromal lymphopoietin (TSLP), IL-33, and IL-25, act as initiators of type 2 inflammation by activating ILC2 and Th2 cells, driving eosinophil recruitment and cytokine production [17]. Tezepelumab (anti-TSLP) has demonstrated broad efficacy in severe asthma irrespective of baseline eosinophil counts but remains investigational for EGPA [32]. Experimental antibodies targeting IL-33 and IL-25 are in early-phase trials [14,23]. Upstream alarmin inhibition may hold promise, particularly in ANCA-negative, eosinophil-dominant phenotypes, but requires further validation before clinical use.

The IL-4/IL-13 signaling cascade and epithelial-alarmin interactions are illustrated in Figure 6 and Figure 7. In summary, while dupilumab provides meaningful airway benefit, clinicians must balance these effects against the potential for EGPA flare, emphasizing careful surveillance and cross-specialty coordination.

**B-cell/autoantibody axis**

B cells and autoantibodies contribute prominently to EGPA pathogenesis, particularly within ANCA-positive subsets. Rituximab, targeting CD20, is an established component of vasculitis-directed therapy [16,18,33].

Emerging strategies now extend beyond B-cell depletion to include plasma-cell-directed approaches. Daratumumab, an anti-CD38 monoclonal antibody that eliminates plasmablasts and long-lived plasma cells, has demonstrated efficacy in refractory ANCA-associated vasculitis [37] and is currently being investigated as a potential therapeutic option in EGPA.

**Figure 8:**
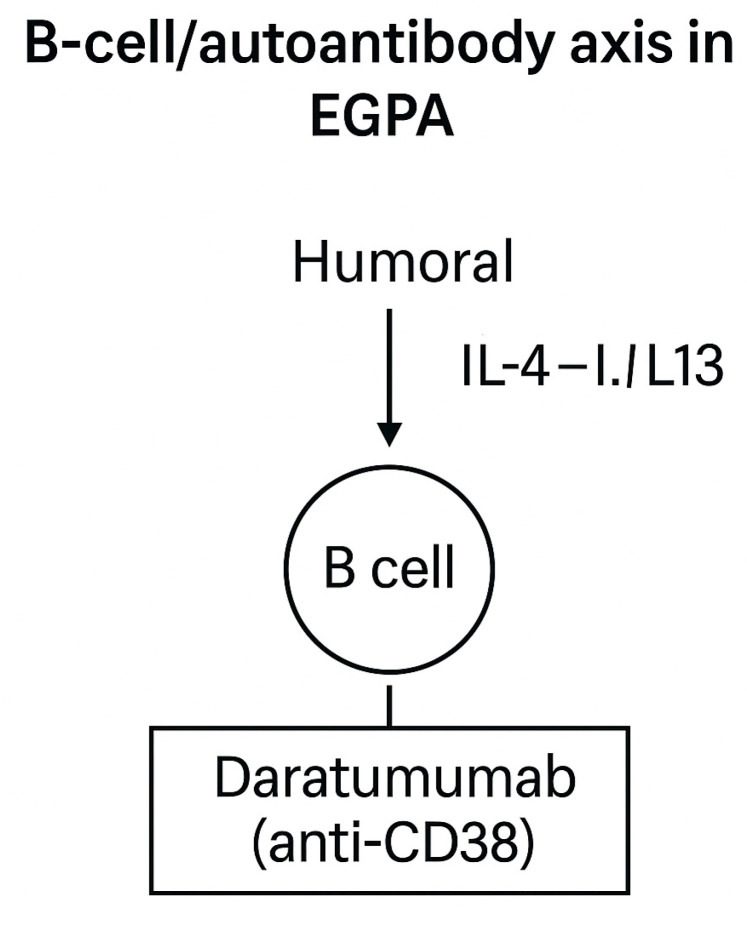
Schematic representation of humoral immune dysregulation in EGPA. B cells contribute to ANCA production and IgG4 responses, supporting vasculitic and Th2-driven mechanisms [13,18]. Beyond rituximab, which targets CD20+ B cells and is part of current standard therapy [16,33], novel strategies are being explored. Daratumumab (anti-CD38) depletes plasmablasts and plasma cells, offering a potential therapeutic option for refractory, autoantibody-driven disease [37].

Although clinical experience remains limited to case-based evidence, this strategy, targeting antibody-producing plasma cells, represents a promising avenue for patients with persistent antibody-mediated activity despite conventional immunosuppression or prior CD20 depletion [16,18,38]. The B-cell/autoantibody axis and related therapeutic targets are illustrated in Figure 8.

**Other emerging strategies**

Addit ional investigational approaches aim to modulate broader inflammatory pathways.

Janus kinase (JAK)/signal transducer and activator of transcription (STAT) inhibitors, such as baricitinib and tofacitinib, block downstream cytokine signaling relevant to type 2 inflammation but require further evaluation of long-term safety, particularly regarding infection and herpes zoster risk [38].

Anti-Siglec-8 antibodies, such as lirentelimab, selectively deplete eosinophils and inhibit mast cell activation, showing encouraging results in eosinophilic disorders and currently under investigation in vasculitis [37]. Complement inhibition with avacopan, an oral C5a receptor antagonist proven effective in GPA and MPA, remains unvalidated in EGPA [23].

Other potential targets include the granulocyte–macrophage colony-stimulating factor (GM-CSF) and interleukin-17 (IL-17) pathways, though their specific roles in EGPA remain to be defined [23].

**Figure 9:**
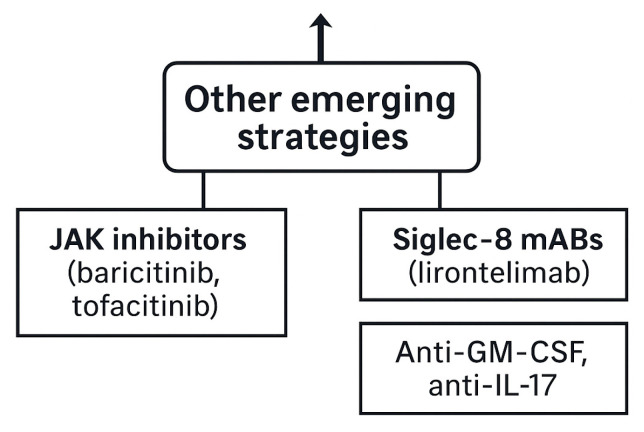
Other emerging strategies. Additional investigational therapies include JAK inhibitors (baricitinib, tofacitinib), Siglec-8 monoclonal antibodies (lirentelimab), and blockade of GM-CSF or IL-17 [23,37,38].

Overall, JAK inhibitors offer theoretical appeal through pathway convergence, while anti-Siglec-8 antibodies demonstrate strong biological plausibility but lack EGPA-specific randomized data. Targeted agents across the IL-5/IL-5Rα, IL-4Rα, epithelial–alarmin, B-cell, and exploratory pathways are summarized in Table 3 and Figure 9.

**Precision-medicine perspective**

Integration of molecular insights with clinical endotyping is reshaping therapeutic strategies in EGPA. IL-5–targeted therapies primarily benefit eosinophil-driven, ANCA-negative disease, whereas B-cell-directed approaches are most effective in ANCA-positive, vasculitic subsets.

Emerging biomarkers—including eosinophil granule proteins, extracellular traps, and multi-omic profiles—are being evaluated to refine patient stratification, guide therapeutic sequencing, and optimize biologic selection [17,21,23].

Ultimately, incorporating these molecular signatures into clinical algorithms may enable adaptive, endotype-specific treatment frameworks, bringing EGPA management closer to true precision medicine.

## 7. Differential Diagnosis and Diagnostic Pitfalls

EGPA must be differentiated from other eosinophilic or vasculitic disorders with overlapping clinical and pathological features. The diagnostic hallmark remains the triad of adult-onset asthma, peripheral eosinophilia, and small- to medium-vessel necrotizing vasculitis, but potential mimickers should be carefully excluded before confirming the diagnosis [1,5,14,23].

Key differential diagnoses

- Hypereosinophilic syndromes (HESs): Persistent eosinophilia >1.5 × 10^9^/L with multi-organ involvement, typically without asthma or ANCA positivity. Bone marrow evaluation and molecular testing for fusion genes such as FIP1L1–PDGFRA help exclude myeloproliferative variants [5,23].
- IgG4-related disease (IgG4-RD): May present with eosinophilia and elevated serum IgG4 but lacks necrotizing vasculitis. Histopathology typically shows storiform fibrosis and dense IgG4^+^ plasma-cell infiltrates. In EGPA, IgG4 elevation reflects Th2 polarization rather than true IgG4-RD [5,14].
- Chronic eosinophilic pneumonia (CEP): Pulmonary infiltrates and marked eosinophilia without systemic vasculitis or extrapulmonary involvement [5,9].
- Parasitic eosinophilia: Travel or exposure history should prompt stool and serologic testing for helminthic infection prior to initiating immunosuppression [23,39].
- Drug-induced eosinophilia or vasculitis: Most frequently associated with leukotriene receptor antagonists, antiepileptics, and antibiotics. Establishing a temporal relationship between drug exposure and symptom onset is key for differentiation [5,39].

Practical diagnostic checklist [1,5,23]:

- Confirm adult-onset asthma and blood eosinophilia (>1.0 × 10^9^/L).
- Exclude infectious, drug-related, or neoplastic causes of eosinophilia.
- Evaluate for vasculitic involvement (neuropathy; renal, skin, or pulmonary capillaritis).
- Test for MPO-ANCA and interpret IgG4 levels contextually.
- When feasible, obtain biopsy demonstrating eosinophilic inflammation and necrotizing vasculitis.
- A systematic, stepwise diagnostic approach integrating clinical, serologic, and histopathologic findings is essential to avoid misclassification and to guide appropriate immunosuppressive or biologic therapy [1,5,14,23].

## 8. Prognosis, Disease Course, and Precision-Therapy Outlook

**Prognosis and disease course by clinical–molecular phenotype**

Prognosis in EGPA reflects the convergence of disease endotype (ANCA-defined), organ involvement, and baseline prognostic indices. The Five-Factor Score (FFS), developed by the French Vasculitis Study Group and validated in EGPA, remains the most practical tool for estimating mortality and guiding therapeutic intensity [40].

The 2009 revised FFS includes five items: age > 65 years, cardiac involvement, gastrointestinal involvement, renal insufficiency (creatinine > 150 µmol/L), and absence of ear–nose–throat manifestations. An FFS ≥ 1 identifies patients at increased risk of death or relapse, in whom systemic immunosuppressants beyond glucocorticoids are recommended [22,36,40].

Importantly, FFS and ANCA-defined endotype complement each other:

ANCA-positive (vasculitic) patients more often exhibit renal and neurologic disease and relapse-driven morbidity.

ANCA-negative (eosinophilic) patients more frequently present with cardiac and gastrointestinal involvement, which are key FFS determinants and leading causes of mortality [6,7,24].

Routine cardiac evaluation, including ECG and echocardiography at baseline and follow-up, with cardiac MRI when clinically indicated, is recommended in all EGPA patients, particularly in ANCA-negative subsets or those with elevated troponin or NT-proBNP [7,24,29]. Emerging data suggest that integrating FFS with molecular biomarkers (e.g., eosinophil-derived proteins, IL-5, proteomic panels) and endotype signatures may enable individualized risk prediction and monitoring in future studies.

**Conceptual framework**

EGPA outcomes are shaped by the interplay between ANCA-defined endotypes and organ-dominant phenotypes. ANCA-positive disease follows a vasculitic trajectory (renal, neuropathic, cutaneous, pulmonary capillaritis) with higher relapse propensity, whereas ANCA-negative disease follows an eosinophilic trajectory (cardiac, pulmonary infiltrates, gastrointestinal) with a greater risk of irreversible organ damage, especially cardiac damage [5,24].

Prognostication is refined by combining the Five-Factor Score, with biomarkers reflecting type-2 inflammation and B-cell/autoantibody activity [21,22,23,39,40].

- ANCA-positive (vasculitic) phenotype: Course and risks: More frequent relapses and renal involvement: neuropathy and purpura are typical: mortality often relates to vasculitic flares or treatment toxicity rather than cardiac failure [5,11,16,22,28,41]. Therapeutic implications: B-cell-directed therapy (rituximab) is effective for induction or relapse and represents an alternative to cyclophosphamide, aligning with the autoantibody-driven biology. IL-5-targeted agents may support asthma or eosinophilia control but are insufficient for severe vasculitis.
- ANCA-negative (eosinophilic) phenotype: Course and risks: Cardiac involvement (eosinophilic myocarditis/cardiomyopathy) drives morbidity and mortality: gastrointestinal disease and migrating pulmonary infiltrates are common: relapses are less common but damage accrues from eosinophil-mediated inflammation [26,30]. Therapeutic implications: IL-5/IL-5Rα blockade (mepolizumab, benralizumab, reslizumab) reduces relapses and glucocorticoid exposure [33,34,35]. Upstream alarmin blockade (tezepelumab) is under investigation for barrier-driven, ANCA-negative trajectories [38].

**Shared airway phenotype (asthma/CRSwNP)**

Asthma (>90%) and chronic rhinosinusitis with nasal polyps (60–80%) are nearly universal and usually precede systemic disease [5,28,40]. Dupilumab (anti-IL-4Rα) effectively controls severe asthma and CRSwNP and may improve upper and lower airway manifestations in EGPA; however, vigilance for vasculitic flares is advised in ANCA-positive subsets [42].

**Biomarkers and risk stratification**

- Type-2 axis: Blood eosinophils, eosinophil cationic protein (ECP), eosinophil-derived neurotoxin (EDN), TARC/CCL17, and EETosis-related markers track eosinophilic activity but incompletely capture vasculitic risk [21,22,23,28].
- Humoral axis: MPO-ANCA identifies the vasculitic endotype and correlates with glomerulonephritis and neuropathy, though titers are imperfect for activity monitoring [5,28,40].
- Multi-omics: Proteomic signatures (SAA1, FGA, SAP, CETP) and integrated biomarker panels can discriminate activity from remission and may inform targeted-therapy allocation [22,23].

These tools, combined with FFS and clinical endotyping, support a layered approach to precision prognostication in EGPA.

**Precision-therapy perspective**

A phenotype-to-target alignment provides a pragmatic framework for long-term management and outcome optimization. A comparative summary of major EGPA phenotypes, associated risks, biomarker profiles, preferred therapeutic targets, and clinical goals is provided in Table 4.

## 9. Research Agenda and Future Directions

Future research in EGPA should focus on translating molecular discoveries into clinically actionable strategies. Key priorities include the following:

- **Validated activity biomarkers:** Development of standardized assays for eosinophil extracellular traps (EETs), eosinophil granule proteins, and proteomic panels to complement BVAS and organ-specific assessment.
- **Prospective, endotype-guided clinical trials:** Stratification of patients by ANCA status and molecular signature to define optimal biologic use and sequencing (IL-5/IL-5Rα, IL-4Rα, B-cell-targeted, JAK/Siglec-8).
- **Standardized cardiac screening:** Implementation of structured ECG–echocardiography–CMR algorithms for early detection and longitudinal monitoring of cardiac involvement, particularly in ANCA-negative EGPA.
- **Complement and B-cell pathways:** Evaluation of complement inhibition (e.g., avacopan) and next-generation B-cell-directed agents (e.g., anti-CD38, anti-BAFF) in refractory or ANCA-positive subsets.
- **Comparative effectiveness and real-world studies:** Head-to-head biologic trials using harmonized endpoints for remission, relapse, and glucocorticoid-sparing outcomes.

Integration of clinical, molecular, and imaging data through multicenter registries will be pivotal for advancing personalized care and refining treatment algorithms in EGPA.

## 10. Discussion and Future Perspectives

Over the past decade, major advances have deepened our understanding of EGPA immunopathogenesis, bridging the gap between clinical phenotypes and molecular mechanisms. Recognition of the two principal endotypes, ANCA-positive (vasculitic) and ANCA-negative (eosinophilic), has provided a framework for individualized management and rational trial design [6,10].

Beyond genetic and immunologic factors, environmental exposures may also contribute to disease initiation within the ANCA-associated vasculitis spectrum. A systematic mapping review identified associations with silica, solvents, pesticides, and smoking, although evidence remains heterogeneous and causality uncertain [42].

Progress in molecular profiling has identified candidate biomarkers and therapeutic targets that may inform precision medicine, although external validation remains limited. The integration of multi-omic data with clinical and imaging findings represents the next step toward personalized risk stratification [22,23].

The therapeutic landscape has evolved substantially. While systemic glucocorticoids remain the cornerstone of treatment, biologic agents targeting IL-5 (mepolizumab, benralizumab, reslizumab) and IL-4/IL-13 (dupilumab) have markedly improved disease control and reduced corticosteroid exposure. Ongoing studies are exploring upstream epithelial–alarmin blockade (tezepelumab), B-cell depletion (rituximab), and emerging JAK and Siglec-8 inhibitors, underscoring the shift toward mechanism-based therapy [16,26,35,37,38,39,42].

Despite this progress, key challenges persist: the scarcity of randomized controlled trials (MIRRA remains the only pivotal RCT), the predominance of observational data, and the limited translation of molecular findings into clinical algorithms. Future efforts must address these gaps through focused mechanistic and interventional research.

**Complement pathway modulation**

Avacopan, an oral C5a receptor antagonist approved for granulomatosis with polyangiitis and microscopic polyangiitis, achieves glucocorticoid-sparing disease control in randomized trials outside EGPA and may be relevant for MPO-ANCA-positive vasculitic phenotypes [43]. Dedicated EGPA trials are still lacking: its use should therefore be considered exploratory pending prospective validation. Beyond C5aR1, upstream inhibitors of C3 and C5 components are under investigation and could broaden complement-directed approaches in systemic vasculitis.

**Tolerogenic and cellular therapies**

Early-phase studies using tolerogenic dendritic cells, regulatory CAR-T cells, and plasma-cell-depleting agents (e.g., anti-CD38 antibodies such as daratumumab) suggest the feasibility of durable immune modulation and antigen-specific tolerance restoration [28,39]. Although still experimental, these strategies hold promise for long-term remission maintenance and immune reprogramming in refractory EGPA.

**Biomarker-guided adaptive algorithms**

Integration of multi-omic signatures, including proteomic, transcriptomic, and metabolomic data, with AI-driven predictive models may enable real-time disease activity monitoring and patient stratification. In the future, biomarker-guided adaptive algorithms could replace static treatment protocols, allowing dynamic adjustment of therapy intensity and sequencing based on evolving molecular activity profiles [22,23]

**Innate immunity modulation**

Direct targeting of epithelial–alarmin circuits, ILC2 activation, and OX40/OX40L signaling represents a promising upstream approach to prevent disease initiation and tissue priming in eosinophilic and barrier-dominant EGPA subsets [23,39]

**Combination and sequencing strategies**

Rational therapeutic combinations, such as IL-5/IL-5Rα blockade with B-cell or complement inhibition, are under conceptual evaluation for mixed or refractory phenotypes [16,23,43]. Future clinical trials must define optimal sequencing, switching criteria, and long-term safety of biologic combinations to achieve sustained remission with minimal glucocorticoid exposure.

**Pediatric and transitional EGPA**

Although rare, pediatric EGPA poses unique diagnostic and therapeutic challenges. Extrapolation from adult paradigms should be cautious, and pediatric-specific registries and clinical trials are urgently needed to establish age-appropriate strategies and ensure smooth transition to adult care.

**Outlook**

The next phase of EGPA research will rely on integrating molecular endotyping, organ-specific risk assessment, and longitudinal multi-omic monitoring to refine personalized therapy, reduce treatment toxicity, and ultimately achieve steroid-free sustained remission [23,24].

## 11. Conclusions

EGPA is a rare, heterogeneous vasculitis in which polygenic susceptibility, type-2 inflammation, eosinophil effector mechanisms, and B-cell/autoantibody responses converge to define distinct clinical and molecular subsets.

Advances in genomics, immunology, and multi-omics have redefined EGPA as a disease that can no longer be approached with uniform therapy.

ANCA-positive patients typically follow a vasculitic trajectory and respond best to B-cell-directed therapies.

ANCA-negative, eosinophil-dominant patients benefit preferentially from IL-5/IL-5Rα blockade, while airway-predominant disease may improve with IL-4Rα inhibition.

The integration of biomarkers, molecular endotyping, and targeted biologics is progressively transforming EGPA into a model for precision medicine in systemic vasculitis. However, challenges remain, including limited randomized data, lack of validated biomarkers, and the management of refractory or overlapping phenotypes.

Continued collaborative efforts across registries, translational studies, and multicenter trials will be essential to close these gaps. Ultimately, aligning therapeutic strategies with molecular and clinical disease drivers offers the best opportunity to improve long-term outcomes, reduce glucocorticoid toxicity, and deliver individualized care for patients with EGPA.

Key Clinical–Molecular Concepts

- Two major endotypes: MPO-ANCA-positive (vasculitic) vs. ANCA-negative (eosinophilic), with distinct organ risks [10,15].
- Genetics segregate endotypes: HLA-DQ (MPO-ANCA+) vs. non-HLA loci (e.g., TSLP, BCL2L11, CDK6, BACH2, LPP, GPA33, IRF1/IL5) in ANCA-negative disease [11,12,13].
- Pathogenesis converges on epithelial–alarmin/type-2 axes and B-cell/autoantibody networks [15,18].
- Biomarkers remain investigational: none replaces BVAS or organ-specific imaging [21,22,23].
- Therapy aligns with biology: rituximab ± cyclophosphamide for vasculitic flares: IL-5/IL-5Rα blockade for eosinophil-dominant disease [16,27,29,34].
- Mixed phenotypes occur and often require combination or sequencing strategies [23,28].
- Complement inhibition, tolerogenic and cellular approaches, and biomarker-guided adaptive algorithms are emerging as next-generation strategies for precision medicine in EGPA [43].

**Practice Points**

- EGPA comprises two major endotypes, ANCA-positive (vasculitic) and ANCA-negative (eosinophilic), with distinct clinical trajectories and risks.
- Mepolizumab and benralizumab are now approved biologics for EGPA, supporting steroid-sparing management.
- ANCA-positive disease often requires B-cell-directed therapy (rituximab) for vasculitis control.
- Baseline and interval cardiac evaluation (ECG, echocardiography, consider CMR) is essential, especially in ANCA-negative subsets.
- Dupilumab may benefit airway-dominant disease (asthma/CRSwNP) but requires vigilance for vasculitic flare.
- Remission should be defined using BVAS and steroid-dose thresholds.
- Ongoing monitoring of eosinophil counts and organ-specific imaging remains central to management.

**Research Agenda**

- Validate EETosis- and omics-based biomarkers for activity and remission.
- Conduct endotype-guided clinical trials integrating genetic and immunologic profiles.
- Standardize cardiac screening and monitoring protocols for EGPA.
- Evaluate complement inhibition and B-cell-targeted strategies in defined subgroups.
- Perform head-to-head biologic comparisons using harmonized endpoints.

## Figures and Tables

**Table 1:** Key clinical contrasts between ANCA-defined endotypes in EGPA. Comparative summary of the two principal EGPA endotypes based on ANCA status. ANCA-positive disease follows a vasculitic trajectory, whereas ANCA-negative disease is driven by eosinophilic inflammation, with distinct organ tropism, biomarker profiles, and therapeutic implications.

Feature	ANCA-Positive (Vasculitic)	ANCA-Negative (Eosinophilic)
Immunopathology	MPO-ANCA-mediated vasculitis	Eosinophil- and barrier-driven inflammation
Predominant organs	Kidney, peripheral nerves, skin, alveolar capillaritis	Heart, lungs (infiltrates), gastrointestinal tract
Relapse pattern	Frequent relapses	Less frequent, but cumulative damage
Biomarker profile	MPO-ANCA+, lower eosinophil counts	ANCA–, higher eosinophil counts, elevated IL-5
Therapeutic emphasis	Rituximab ± cyclophosphamide (vasculitic phenotype)	IL-5/IL-5Rα blockade (mepolizumab, benralizumab)
Prognostic concern	Renal and vasculitic flares	Cardiac involvement and fibrosis

**Table 2:** Conventional therapies in EGPA. Overview of the main drug classes historically and currently used in the management of EGPA. Glucocorticoids remain the cornerstone of induction therapy, while conventional immunosuppressants (cyclophosphamide, azathioprine, methotrexate, mycophenolate mofetil) are employed as induction or maintenance agents depending on disease severity [3,23,29]. Intravenous immunoglobulin (IVIG) may be considered in refractory cases, particularly for neuropathy [35], and rituximab is used in ANCA-positive patients with vasculitic features [16,33]. Anti-IL-5 and anti-IL-5Rα biologics have expanded treatment options: mepolizumab and benralizumab are now both approved for EGPA, following the MIRRA and MANDARA phase 3 trials, respectively [27,28,36]. In MANDARA, benralizumab (anti-IL-5Rα) demonstrated non-inferior remission rates versus mepolizumab (59% vs. 56% at weeks 36 and 48), with comparable safety and steroid-sparing benefit [28]. Benralizumab’s antibody-dependent eosinophil depletion distinguishes its mechanism from IL-5 neutralization. Reslizumab may be considered off-label in selected cases with high eosinophil burden or airway-dominant disease [23,30]. Despite these advances, relapses, cumulative glucocorticoid toxicity, and the absence of universal biomarkers highlight the ongoing need for precision-medicine approaches [23].

Drug/Class	Indication/Use	Comments
Glucocorticoids	First-line: induction of remission: tapered for maintenance	High initial response: frequent relapses: long-term toxicity: ensure bone and infection prophylaxis.
Cyclophosphamide	Severe, organ- or life-threatening disease (renal, pulmonary hemorrhage, neuropathy)	Potent but toxic: used in acute severe presentations or refractory vasculitic disease.
Azathioprine	Maintenance therapy after remission induction: less severe cases	Steroid-sparing: moderate efficacy: requires toxicity and blood count monitoring.
Methotrexate	Maintenance therapy: steroid-sparing agent	Alternative for maintenance: data extrapolated from other AAV.
Mycophenolate mofetil	Alternative maintenance therapy: sometimes in refractory cases	Used when intolerance or contraindication to other agents.
IVIG	Adjunctive therapy in refractory disease, especially neuropathy	Evidence limited to small series: immunomodulatory effect.
Rituximab	ANCA-positive EGPA: vasculitic phenotype: refractory cases	Established in GPA/MPA: efficacy in EGPA mainly in ANCA-positive subset: suitable for induction or relapse.
Mepolizumab (anti-IL-5)	Approved biologic: relapsing/refractory EGPA: steroid-sparing	Only biologic formally approved for EGPA (MIRRA trial): reduces relapses and glucocorticoid dependence.
Benralizumab (anti-IL-5Rα)	Approved biologic for adult EGPA: eosinophil-dominant disease: relapse prevention: steroid-sparing	Head-to-head phase 3 MANDARA trial showed non-inferior remission vs. mepolizumab with Q4W dosing: mechanism: IL-5Rα binding with ADCC-mediated eosinophil depletion: consider in high eosinophil burden or inadequate response to IL-5 blockade: monitor glucocorticoid taper per protocol.

**Table 3:** Targeted and emerging therapies in eosinophilic granulomatosis with polyangiitis (EGPA): pathway alignment and evidence level. This table summarizes key biologic and investigational targets according to the immunologic pathway, including their clinical maturity and supporting evidence. Evidence levels are defined as follows: high (randomized controlled or real-world data), moderate (off-label or disease-extrapolated data), and exploratory (early-phase or preclinical data). CRSwNP = chronic rhinosinusitis with nasal polyps: RCT = randomized controlled trial; FDA = U.S. Food and Drug Administration: EMA = European Medicines Agency. (Adapted from [17,23]).

Pathway/Target	Therapies/Agents	Notes	Evidence Level/Development Stage
IL-5 axis	Mepolizumab (anti-IL-5), Reslizumab (anti-IL-5), Benralizumab (anti-IL-5Rα)	Most validated: reduces relapses and steroid dependence: effective in eosinophilic/ANCA-negative disease	High: RCT (MIRRA, MANDARA) and real-world cohorts: FDA/EMA-approved for EGPA
IL-4/IL-13 axis	Dupilumab (anti-IL-4Rα)	Effective in asthma/CRSwNP: case reports in EGPA: risk of vasculitic flares not excluded	Moderate: Approved for asthma/CRSwNP: off-label in EGPA: case-based evidence
Epithelial alarmins	Tezepelumab (anti-TSLP): experimental blockade of IL-33 and IL-25	Upstream blockade: promising for ANCA-negative phenotypes: under investigation	Moderate: Approved for asthma/CRSwNP: off-label in EGPA: case-based evidence
B-cell/Autoantibody axis	Daratumumab (anti-CD38: plasmablast/plasma cell depletion)	Emerging strategy: anecdotal evidence in refractory ANCA-associated vasculitis: rationale for autoantibody-driven EGPA	High (RTX) RCT/observational in AAV and EGPA: Exploratory (Daratumumab) case reports only
Other emerging strategies	JAK inhibitors (baricitinib, tofacitinib): Siglec-8 mAbs (lirentelimab): anti-GM-CSF: anti-IL-17	Investigational: promising but not yet validated in EGPA	Exploratory: Early-phase or preclinical data: not yet validated in EGPA

**Table 4:** Prognosis and targeted therapy by phenotype/endotype in EGPA. Clinical outcomes in EGPA vary by phenotype: ANCA+ disease shows vasculitic relapses and responds to B-cell-directed therapy [11,16,23]: ANCA– disease carries higher cardiac risk, where IL-5/IL-5Rα blockade is most effective [23,26,28,30]: airway-dominant disease (asthma/CRSwNP) benefits from IL-4Rα inhibition [31]: and refractory or mixed phenotypes may require emerging strategies [37,39].

Phenotype/Endotype	Main Risks	Biomarkers/Features	Preferred Therapeutic Targets	Clinical Goal	Notes
ANCA-positive (vasculitic)	Systemic vasculitis flares: GN: neuropathy: relapse propensity	MPO-ANCA^+^: lower eosinophils vs. ANCA–: purpura: mononeuritis multiplex	B-cell axis → Rituximab (±CYC for organ-/life-threatening): IL-5 axis adjunct for airway/eosinophils	Prevent relapses: preserve renal/neurologic function: minimize GC exposure	Treat vasculitis first: manage airway disease in parallel
ANCA-negative (eosinophilic)	Cardiac damage (eosinophilic myocarditis/cardiomyopathy): GI disease: pulmonary infiltrates	High eosinophils: epithelial/mucosal signature:IL5/TSLP/GPA33 genetics	IL-5/IL-5Rα → Mepolizumab, Benralizumab (±Reslizumab): consider TSLP blockade (tezepelumab)	Reduce eosinophilic activity: protect heart: achieve GC-sparing	Early cardiac screening and follow-up are critical
Airway-dominant (asthma/CRSwNP)	Exacerbations: steroid dependence: QoL impairment	Type 2 profile: FeNO↑: eosinophils: CRSwNP present	IL-4Rα → Dupilumab: IL-5/IL-5Rα for eosinophilic burden: optimize inhaled/topical therapy	Control airway disease: reduce exacerbations and OCS: improve QoL	Monitor for vasculitic flares in ANCA^+^ while optimizing airway control
Refractory/mixed phenotype	Persistent activity despite standard biologic: cumulative toxicity	Overlapping vasculitic and eosinophilic signatures: biomarker heterogeneity	Clinical trials/emerging: JAK inhibitors: Siglec-8 mAbs: anti-GM-CSF: anti-IL-17: consider RTX re-treatment	Achieve disease control with steroid sparing: address dominant pathway	Biomarker-guided and endotype-driven adjustments recommended

## Data Availability

No new data were created or analyzed in this study. Data sharing is not applicable to this article.
